# Identification and Characterization of Four Autophagy-Related Genes That Are Expressed in Response to Hypoxia in the Brain of the Oriental River Prawn (*Macrobrachium nipponense*)

**DOI:** 10.3390/ijms20081856

**Published:** 2019-04-15

**Authors:** Shengming Sun, Ying Wu, Hongtuo Fu, Xianping Ge, Hongzheng You, Xugan Wu

**Affiliations:** 1 Wuxi Fishery College, Nanjing Agricultural University, Wuxi 214081, China; sunsm@ffrc.cn (S.S.); wuying@ffrc.cn (Y.W.); 2 Key Laboratory of Freshwater Fisheries and Germplasm Resources Use, Ministry of Agriculture, Freshwater Fisheries Research Center, Chinese Academy of Fishery Sciences, Wuxi 214081, China; gexp@ffrc.cn; 3 Tianjin Fisheries Research Institute, Tianjin 300221, China; yy_8245@163.com; 4 Key Laboratory of Exploration and Use of Aquatic Genetic Resources, Shanghai Ocean University, Shanghai 201306, China; xgwu@shou.edu.cn

**Keywords:** Autophagy, Hypoxia, *Macrobrachium nipponense*, Brain, RNA interference

## Abstract

Autophagy is a cytoprotective mechanism triggered in response to adverse environmental conditions. Herein, we investigated the autophagy process in the oriental river prawn (*Macrobrachium nipponense*) following hypoxia. Full-length cDNAs encoding autophagy-related genes (ATGs) *ATG3*, *ATG4B*, *ATG5*, and *ATG9A* were cloned, and transcription following hypoxia was explored in different tissues and developmental stages. The *ATG3*, *ATG4B*, *ATG5*, and *ATG9A* cDNAs include open reading frames encoding proteins of 319, 264, 268, and 828 amino acids, respectively. The four *M. nipponense* proteins clustered separately from vertebrate homologs in phylogenetic analysis. All four mRNAs were expressed in various tissues, with highest levels in brain and hepatopancreas. Hypoxia up-regulated all four mRNAs in a time-dependent manner. Thus, these genes may contribute to autophagy-based responses against hypoxia in *M. nipponense*. Biochemical analysis revealed that hypoxia stimulated anaerobic metabolism in the brain tissue. Furthermore, in situ hybridization experiments revealed that *ATG4B* was mainly expressed in the secretory and astrocyte cells of the brain. Silencing of *ATG4B* down-regulated *ATG8* and decreased cell viability in juvenile prawn brains following hypoxia. Thus, autophagy is an adaptive response protecting against hypoxia in *M. nipponense* and possibly other crustaceans. Recombinant *MnATG4B* could interact with recombinant *MnATG8*, but the *GST* protein could not bind to *MnATG8*. These findings provide us with a better understanding of the fundamental mechanisms of autophagy in prawns.

## 1. Introduction

Hypoxia is a natural phenomenon in aquaculture ponds, especially in summer, that negatively impacts aquatic animals by limiting behavior, growth, reproduction and even survival [1]. Oriental river prawn (*Macrobrachium nipponense*) is a useful test organism for laboratory-based hypoxia research because it is small and reproduces rapidly. Recent studies in our laboratory showed that hypoxia causes energy metabolism disorders in *M. nipponense* brain tissue [2]. Although these previous studies revealed up-regulation of the autophagic/lysosomal pathway during hypoxia stress [3], the molecular components and regulatory networks in crustaceans have not yet been reported. Given the high conservation of the autophagic machinery [4,5,6], it is reasonable to hypothesize that autophagy represents an adaptive response to hypoxia stress in prawns.

Transportation of cytoplasmic cargo to lysosomes is dependent on autophagy since this process contributes to homeostasis of organelles and proteins [7]. Autophagy involves autophagy-related genes (*ATGs*) present in yeast and higher eukaryotes [8], but only a few *ATGs* have been cloned and characterized from fish, including *Beclin1* in *Gobiocypris rarus* [9], *Paralichthys olivaceus* [10] and common carp (*Cyprinus carpio*) [11], *ATG5* in *Danio rerio* [12] and *ATG4* in *Pelteobagrus fulvidraco* [13]. A recent study identified ATGs in *Macrobrachium rosenbergii* transcriptome data and examined the presence of key ATG proteins in tissues using western blotting [7]. However, other studies on *ATG* mRNA expression profiles have not been reported.

**Figure 1:**
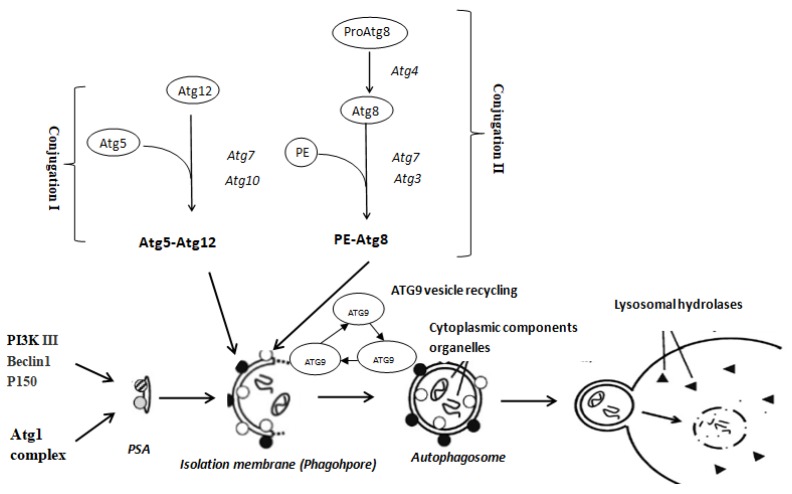
Schematic representation of the autophagy pathway from reference by Seiliez et al. (2010).

During autophagy, regions of the cytoplasm and organelles are sequestered into vacuoles known as autophagosomes [14] that include the *Beclin1/ATG6* complex and *ATG7-ATG3-ATG5* ubiquitin-like conjugation, *MAP1LC3* conjugation and *ATG9* vesicle recycling systems (Figure 1). *ATG3*, *ATG4, ATG5* and *ATG9* are crucial regulatory components of ubiquitin-like modification systems in autophagosomes [15]. *ATG8* is conjugated to the membrane lipid phosphatidylethanolamine by *ATG4/ATG3* and inserted into the autophagosomal membrane by *ATG9* [16,17]. We previously demonstrated that apoptosis can be triggered in the brain following exposure to reactive oxygen species (ROS) caused by hypoxia stress [2], and ROS may also induce the cytoprotective function of autophagy.

In the present study, we investigated autophagy in the brain of *M. nipponense* following hypoxia exposure. We cloned and characterized full-length cDNAs of four ATGs (*ATG3*, *ATG4B*, *ATG5*, and *ATG9A*) reportedly involved in autophagosome expansion and membrane elongation, and explored their expression in different tissues and developmental stages in juvenile prawns, under normal and hypoxia conditions. The findings expand our understanding of autophagy and hypoxia stress at the molecular level, and illuminate the adaptive protective mechanism of autophagic responses to hypoxia in crustaceans.

## 2. Results

### 2.1. Characterization and Phylogenetic Analysis of M. nipponense ATG3, ATG4B, ATG5, and ATG9A.

**Figure 2:**
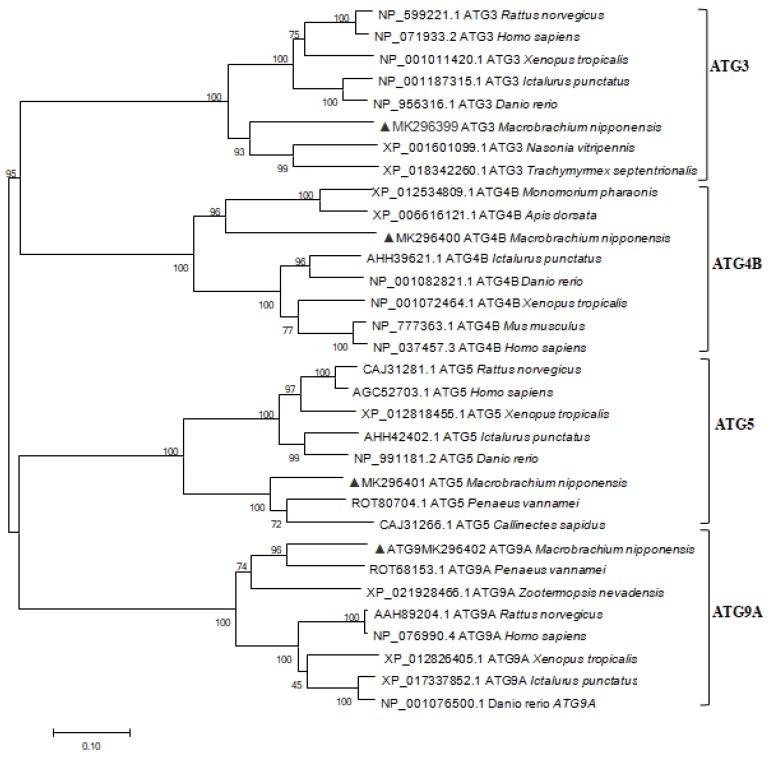
Phylogenetic tree of autophagy-related genes from *M. nipponense* and related organisms constructed using the neighbor-joining method with Molecular Evolutionary Genetics Analysis software version 4.0 (MEGA4) (available online: http://www.megasoftware.net/mega4/mega.html, access date: 12 April 2019). Numbers at branches indicate percentage bootstrap values.

**Figure A1:**
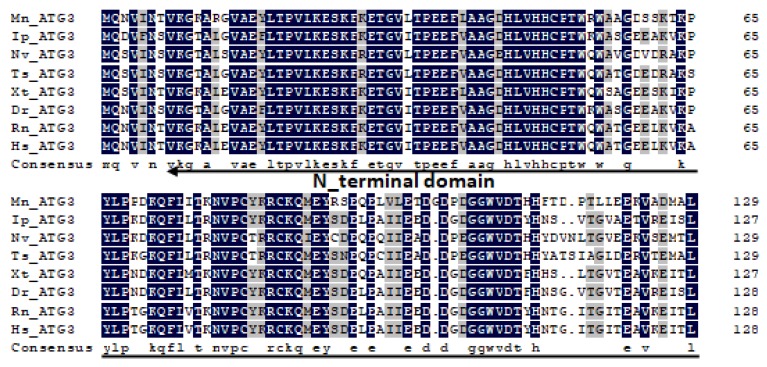

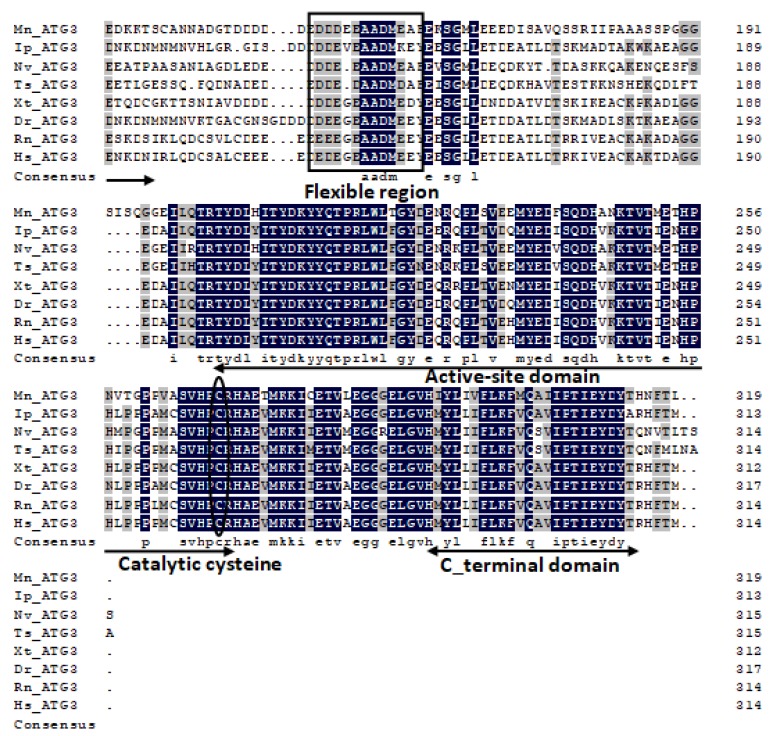
Multiple amino acid sequence alignment of ATG3 from *Macrobrachium nipponensis* and other species. The accession numbers for ATG3 from *Macrobrachium nipponensis* (Mn), *Ictalurus punctatus* (Ip), *Nasonia vitripennis* (Nv), *Trachymyrmex septentrionalis* (Ts), *Xenopus tropicalis* (Xt), *Danio rerio* (Dr), *Rattus norvegicus* (Rn) and *Homo sapiens* (Hs) are MK296399, NP_001187315.1, XP_001601099.1, XP_018342260.1, NP_001011420.1, NP_956316.1, NP_599221.1, and NP_071933.2 respectively. The arrows below the sequences indicate conserved domains (N-terminal domain, C-terminal domain and active-site domain). AA within the oval frame represents the conserved catalytic cysteine. The flexible region (FR) is boxed.

**Figure A2:**
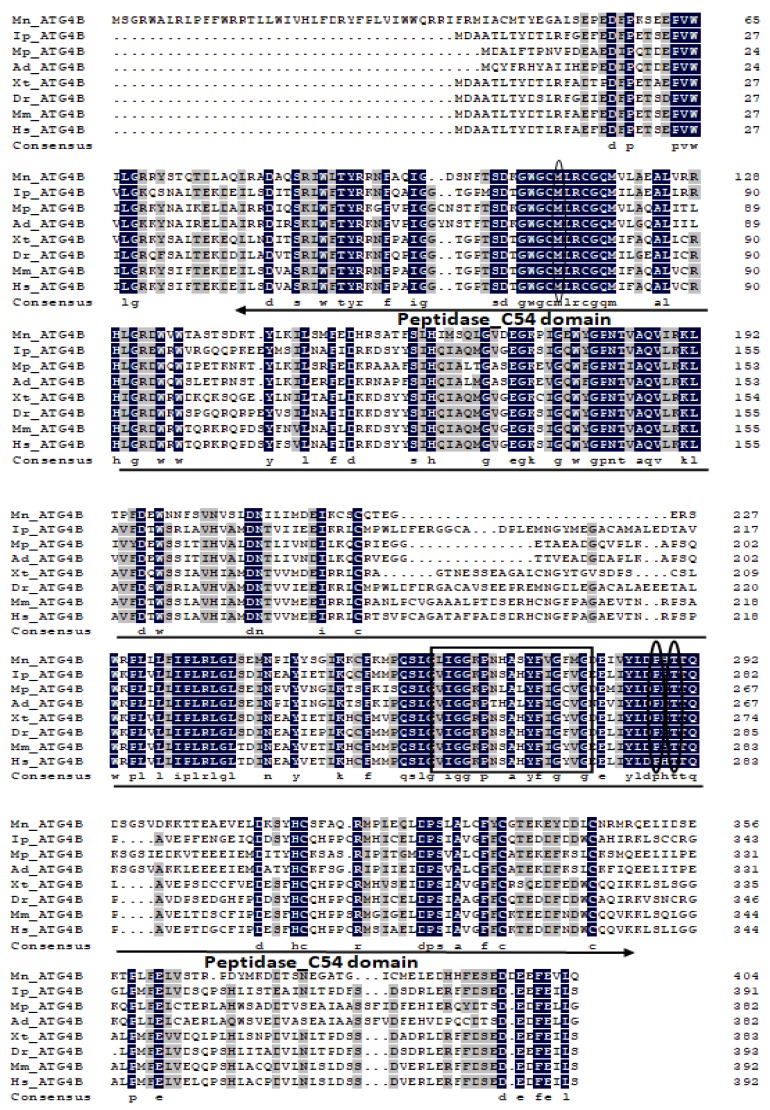
Multiple amino acid sequence alignment of ATG4B from *Macrobrachium nipponensis* and other species. The accession numbers for ATG4B from *Macrobrachium nipponensis* (Mn), *Ictalurus punctatus* (Ip), *Monomorium pharaonis* (Mp), *Apis dorsata* (Ad), *Xenopus tropicalis* (Xt), *Danio rerio* (Dr), *Mus musculus* (Mm) and *Homo sapiens* (Hs) are MK296400, AHH39621.1, XP_012534809.1, XP_006616121.1, NP_001072464.1, NP_001082821.1, NP_777363.1, and NP_037457.3 respectively. The arrows below the sequences represent the Peptidase_C54 domain. AA within the oval frame represents the conserved active catalytic triad sites. The conserved regulatory loop is boxed.

**Figure A3:**
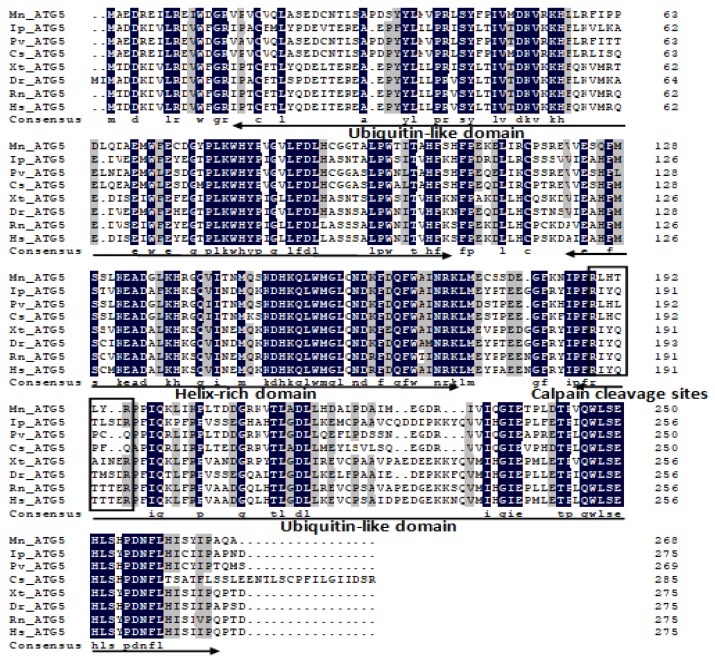
Multiple amino acid sequence alignment of ATG5 from *Macrobrachium nipponensis* and other species. The accession numbers for ATG5 from *Macrobrachium nipponensis* (Mn), *Ictalurus punctatus* (Ip), *Penaeus vannamei* (Pv), *Callinectes sapidus* (Cs), *Xenopus tropicalis* (Xt), *Danio rerio* (Dr), *Rattus norvegicus* (Rn) and *Homo sapiens* (Hs) are MK296401, AHH42402.1, ROT80704.1, CAJ31266.1, XP_012818455.1, NP_991181.2, CAJ31281.1, and AGC52703.1 respectively. The arrows below the sequences indicate the conserved domain (two ubiquitin-like domains and a helix-rich domain). The conserved calpain cleavage sites are boxed.

**Figure A4:**
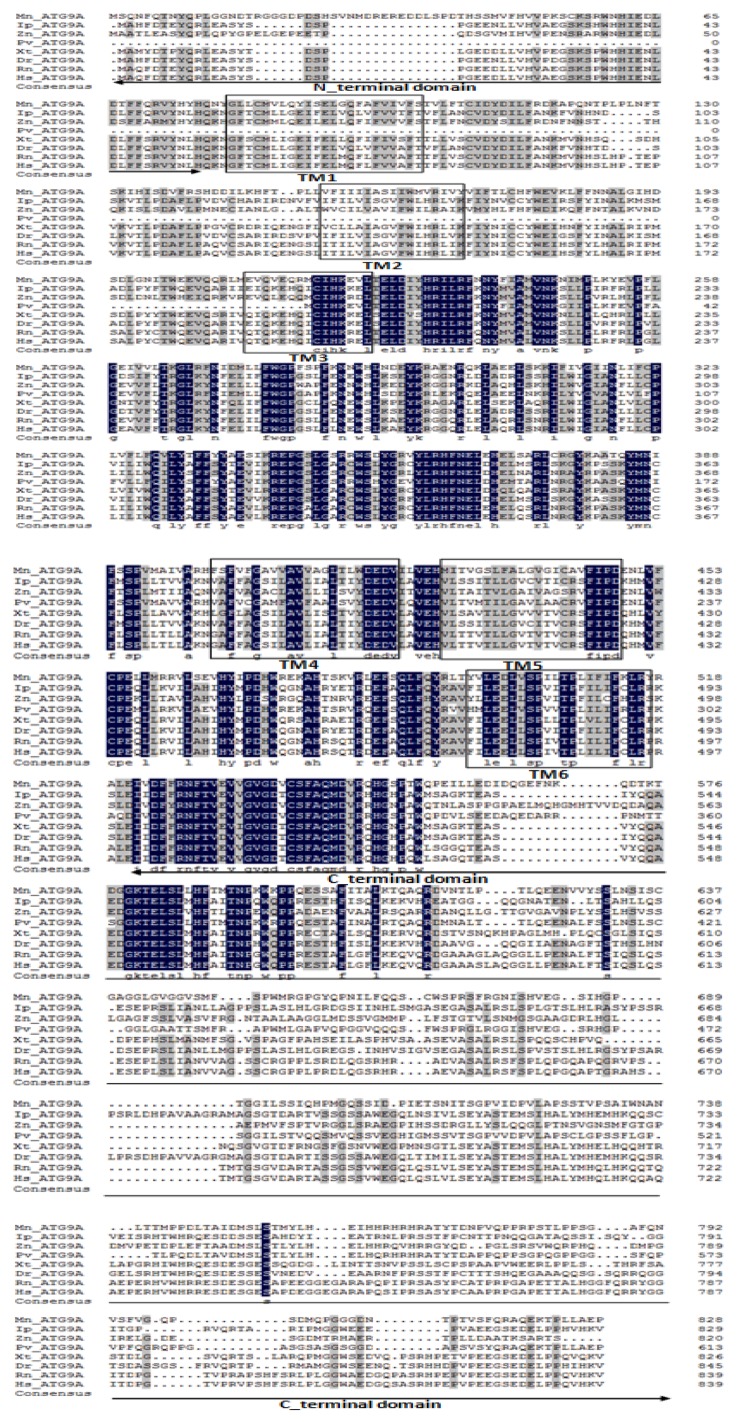
Multiple amino acid sequence alignment of ATG9A from *Macrobrachium nipponensis* and other species. The accession numbers for ATG9A from *Macrobrachium nipponensis* (Mn), *Ictalurus punctatus* (Ip), *Zootermopsis nevadensis* (Zn), *Penaeus vannamei* (Pv), *Xenopus tropicalis* (Xt), *Danio rerio* (Dr), *Rattus norvegicus* (Rn) and *Homo sapiens* (Hs) are MK296402, XP_017337852.1, XP_021928466.1, ROT68153.1, XP_012826405.1, NP_001076500.1, AAH89204.1, and NP_076990.4 respectively. The arrows below the sequences indicate the conserved domains (N-terminal domain and C-terminal domain). The six transmembrane helices (TM) are boxed.

Rapid amplification of the cDNA ends (RACE) of the *ATG3*, *ATG4B*, *ATG5*, and *ATG9A* fragments yielded cDNA sequences of length 1652 bp, 1512 bp, 1731 bp and 3072 bp respectively (GenBank Accession No. MK296399, MK296400, MK296401, and MK296402 respectively), including open reading frames (ORFs) encoding proteins of 319, 264, 268 and 828 amino acids respectively (calculated molecular mass = 35.80 kDa, 47.05 kDa, 31.03 kDa and 94.53 kDa respectively; theoretical isoelectric point [pI] values = 4.65, 5.11, 5.61 and 5.99, respectively). None of the genes were predicted to include a signal peptide according to SignalP 4.0 Server (available online: http://www.cbs.dtu.dk/services/SignalP-4.0/, access date: 12 April 2019). The deduced polypeptide encoded by the *M. nipponense ATG3* cDNA encodes a typical conserved active-site domain and a flexible region (Figure A1). *M. nipponense ATG4B* possesses characteristic features reminiscent of cysteine protease Atg4, including a conserved Peptidase_C54 domain and a probable catalytic Cys residue (Figure A2). *M. nipponense ATG5* contains two ubiquitin-like domains, a helix-rich domain, and conserved calpain cleavage sites (Figure A3). *M. nipponense ATG9A* comprises an N- and C-terminal domains, and six transmembrane helices (Figure A4). Phylogenetic trees were constructed based on the deduced amino acid sequences of the four *ATGs*, revealing their evolutionary relationships (Figure 2), which were in agreement with the traditional taxonomic classification of the included species.

### 2.2. Expression of ATG3, ATG4B, ATG5, and ATG9A in Different Tissues and Developmental Stages

**Figure 3:**
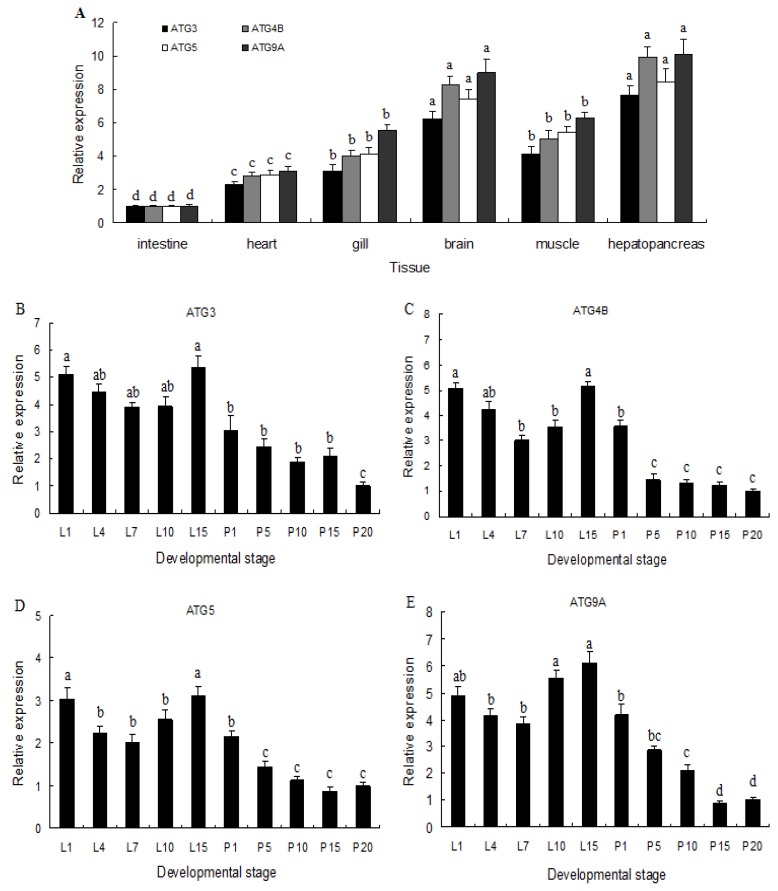
Quantitative real-time PCR (qRT-PCR) analysis of *M. nipponense ATG3*, *ATG4B*, *ATG5*, and *ATG9A* mRNA expression levels in different tissues (**A**) and developmental stages (**B**–**E**). Larvae were collected every 4 days between 1-day post-hatching (L1) and 1 day before metamorphosis (L13). Post-larvae were collected every 5 days between 1 and 20 days after metamorphosis (P1P20), and every 10 days between P20 and P30. Data are presented as mean ± standard error of the mean (SEM) for triplicate samples, and were normalized against the *β-actin* housekeeping gene. Bars sharing different letters within the same gene indicate significant differences among different tissues (*p* < 0.05).

Expression of *M. nipponense ATG3*, *ATG4B*, *ATG5*, and *ATG9A* in different tissues and developmental stage was investigated by qRT-PCR, and mRNAs of all four genes were widely expressed in hepatopancreas, gill, muscle, brain, heart, and intestine, with highest expression in brain and hepatopancreas (Figure 3A). Based on these expression results, brain tissue was chosen for the subsequent experiments. Although transcripts of all four genes were detected during all developmental stages, levels were higher in early and late larval stages (*p* < 0.05) than the post-larval stage (Figure 3B–E). Thus, the present results indicated that *ATG3*, *ATG4B*, *ATG5*, and *ATG9A* are widely expressed in different tissues and developmental stages.

### 2.3. Expression of ATG3, ATG4B, ATG5, and ATG9A Following Hypoxia

**Figure 4:**
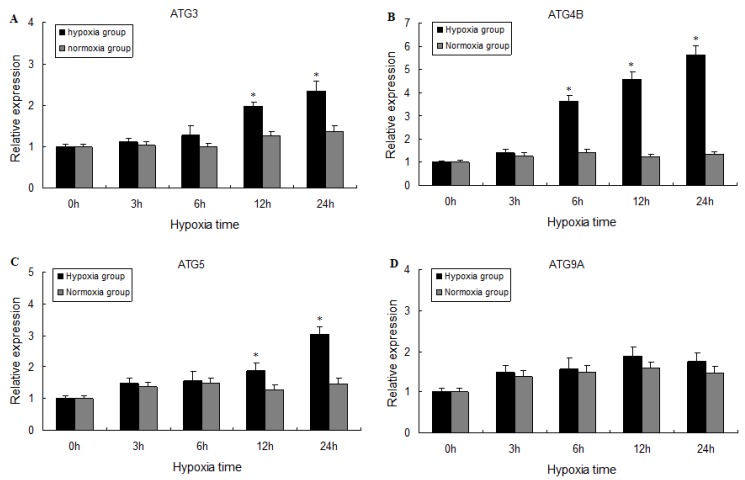
Effects of hypoxia on mRNA expression levels of *ATG3* (**A**), *ATG4B* (**B**), *ATG5* (**C**) and *ATG9A* (**D**) in the brain of *M. nipponense*. Values are presented as mean ± SEM for triplicate samples. Bars with asterisks indicate significant differences between control and hypoxia groups (*p* < 0.05).

To examine whether hypoxia affects *M. nipponense ATG3*, *ATG4B*, *ATG5*, and *ATG9A* mRNA expression, prawns were exposed to controlled hypoxia for 24 h, and mRNA expression levels of all four *ATG* genes increased in proportion to hypoxia duration, reaching maximum levels at 24 h post-exposure (Figure 4A–C). *ATG4B* mRNA expression was significantly higher than controls in brain following hypoxia for 6 h, 12 h and 24 h (*p* < 0.05), whereas *ATG3* and *ATG5* mRNA levels in brain were only slightly elevated (*p* < 0.05) after 12 h and 24 h of hypoxia. However, no significant differences in the *ATG9A* mRNA expression levels were observed (Figure 4D). The results indicate that most autophagy-related genes were expressed in response to the hypoxic challenge.

### 2.4. Localization of ATG4B mRNA in the Brain

**Figure 5:**
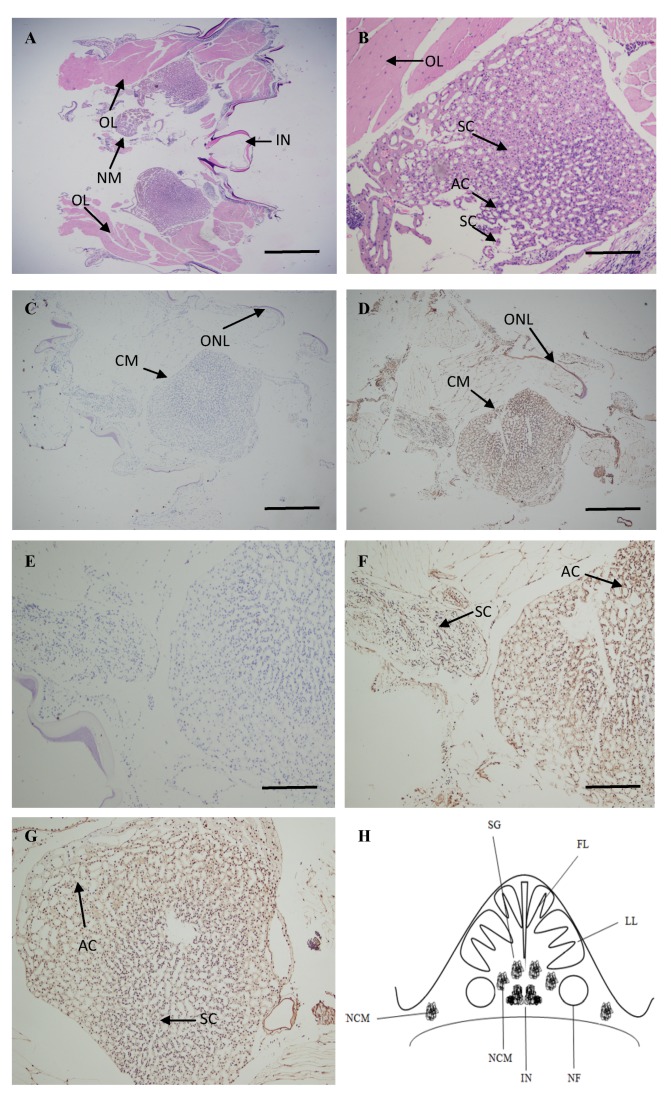
In situ hybridization of *M. nipponense ATG4B* transcripts in the brain tissue. (**A**) Photograph of *M. nipponense* overall brain including the nervous mass (NM), optic lobe (OL) and intermediate neurons (IN). (**B**) Nervous mass of *M. nipponense* showing secretory cells (SCs) and astrocyte cells (ACs). (**C**) and (**E**) Sense probes were used as negative controls. (**D**) *ATG4B* expression in the brain tissue of prawns in the cell mass (CM) and optic nerve lamella (ONL). (**F**) and (**G**) *ATG4B* was mainly expressed in the secretory cells and astrocytes cells of prawns in response to normoxia and hypoxia for 24 h, secretory cells and astrocytes cells have been distinguished with different shape, such as round and oval. (**H**) A schematic of the organization of the prawn brain. SG: spinal ganglion cells, NCM: neural cells mass, IN: intermediate neurons, NF: nerve fibers, FL: frontal lobe, LL: lateral lobes. Scale bar: 20 μm (**A**,**C**,**D**), 50 μm (**B**,**E**,**F**,**G**).

Since the *M. nipponense ATG4B* expression levels were significantly affected by hypoxia, the distribution and localization of *ATG4B* were examined in the brain of *M. nipponense* during normoxia and hypoxia by ISH. The overall brain structure and nervous mass are shown in Figure 5A,B, respectively. The prawn brain is composed of the nervous mass, the optic lobe, and the intermediate neurons. The nervous mass mainly contains two types of cells—secretory cells and astrocytes. No signals were observed in the negative control experiments with the sense strand probe in the overall brain structure and nervous mass (Figure 5C,E). The *ATG4B* transcripts were mainly localized in the nervous mass, and a few *ATG4B* transcripts were localized in the optic lobe under normoxic conditions (Figure 5D). Furthermore, in response to normoxia and hypoxia, a positive signal was obtained for the antisense probe both in the round-shaped secretory cells and oval-shaped astrocytes from the cerebral ganglion (Figure 5F,G). A schematic of the organization of the prawn brain was shown in Figure 5H. Thus, we next investigated the functions of *ATG4B* in brain of prawns in response to hypoxia.

### 2.5. Biochemical Analysis

**Figure 6:**
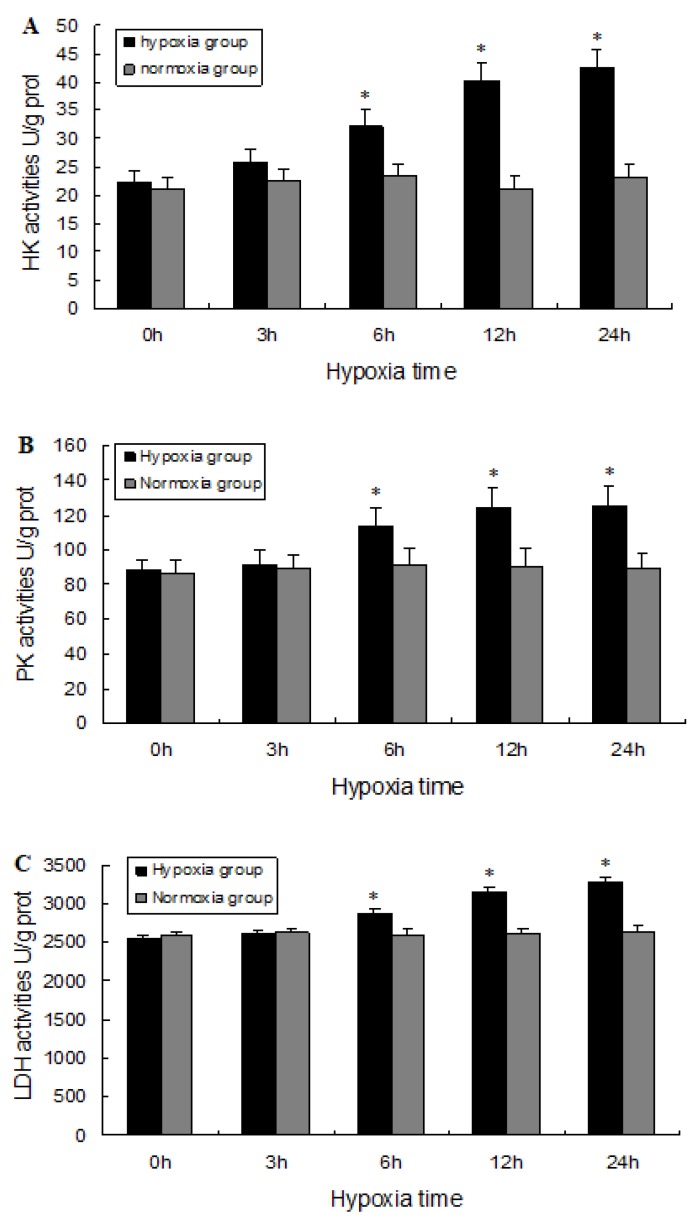
Enzyme activity of hexokinase (HK, **A**), pyruvate kinase (PK, **B**), and lactate dehydrogenase (LDH, **C**) in the brain of prawns in response to hypoxia. Values are presented as mean ± SEM for triplicate samples. Bars with asterisks indicate significant differences between the control and hypoxia groups (*p* < 0.05).

Enzyme activity was analyzed to confirm that hypoxia resulted in alterations in the metabolic pathways in the prawn brain tissue. The activities of hexokinase (HK), pyruvate kinase (PK), and lactate dehydrogenase (LDH) enzymes were significantly higher (*p* < 0.05) in brain after 6 h, 12 h and 24 h of hypoxia than levels in normoxia groups (Figure 6A–C). The findings indicate that hypoxia resulted in an acceleration in anaerobic glycolysis. Thus, metabolic function in the brain of juvenile *M. nipponense* was impaired by acute hypoxia, because the glycolytic pathway as a major source of energy is not sufficient for the ATP supply required. Next, we tried to determine whether autophagy acts as an important cytoprotective mechanism in the brain tissue of prawns in response to hypoxia.

### 2.6. Effect of ATG4B Gene Silencing on ATG8 Expression and Cell Viability in Brain

**Figure 7:**
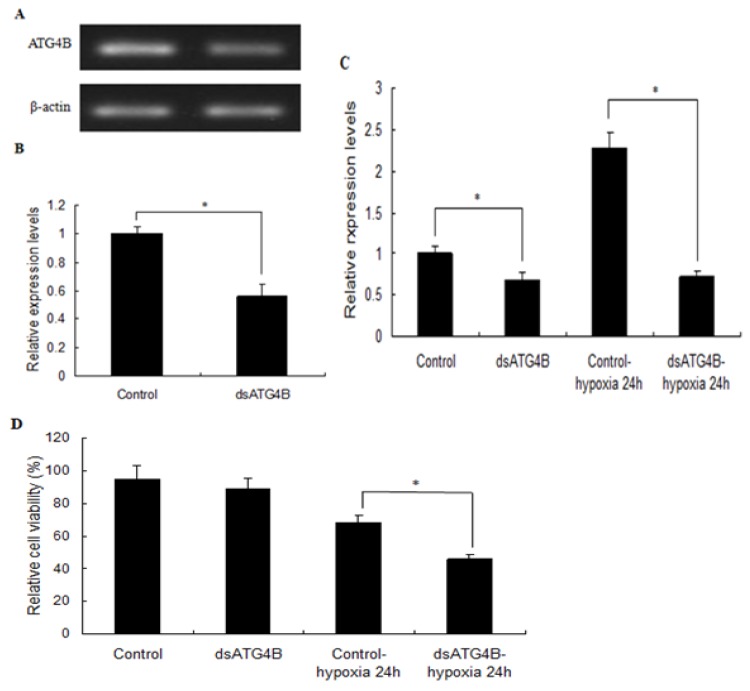
RNA interference (RNAi)-based functional analysis of *ATG4B* in *M. nipponense*. RNAi efficiency of *ATG4B* silencing compared with enhanced green fluorescent protein (eGFP) at 4 days post-dsRNA injection was analyzed by semi-quantitative PCR (**A**) and qRT-PCR (**B**). *ATG8* mRNA expression levels (**C**) and cell viability (**D**) in *M. nipponense* juvenile brains after dsRNA injection were analyzed under hypoxia and normoxia conditions. Results were normalized against *β-actin* as an internal transcription reference. Data are presented as mean ± SE of three independent biological replicates.

To investigate the potential function of autophagy in response to hypoxia in prawns, expression of *ATG4B* in brain was silenced using dsRNA (Figure 7A,B). The ubiquitin-like protein *ATG8* is a reliable marker of the induction and progression of autophagy. Thus, *ATG8* in *M. nipponense* was cloned and its mRNA expression levels were analyzed under hypoxia conditions in our laboratory [3]. The expression of *ATG8* was relatively stable (< 5% changed) in brain tissue of control prawns not injected with dsRNA. By contrast, ATG8 expression decreased by 68% or 72% after 24 h under normoxia or hypoxia conditions in the brains of dsRNA-injected prawns when *ATG4B* is absent (Figure 7C); this indicates that ATG8 expression is regulated by ATG4B, as reported in a previous study on rainbow trout (*Oncorhynchus mykiss*) [15]. Injection of neither ds*ATG4B* nor dsEGFP affected cell viability in *M. nipponense* juveniles prior to hypoxia exposure. However, following *ATG4B* knockdown, there was a significant (*p* < 0.05) reduction in cell viability in *ATG4B*-depleted prawns compared with dsEGFP-injected prawns after 24 h of hypoxia (Figure 7D). Our results provide evidence that autophagy plays a protective role in hypoxic stress.

### 2.7. Pull-down Analysis

**Figure 8:**
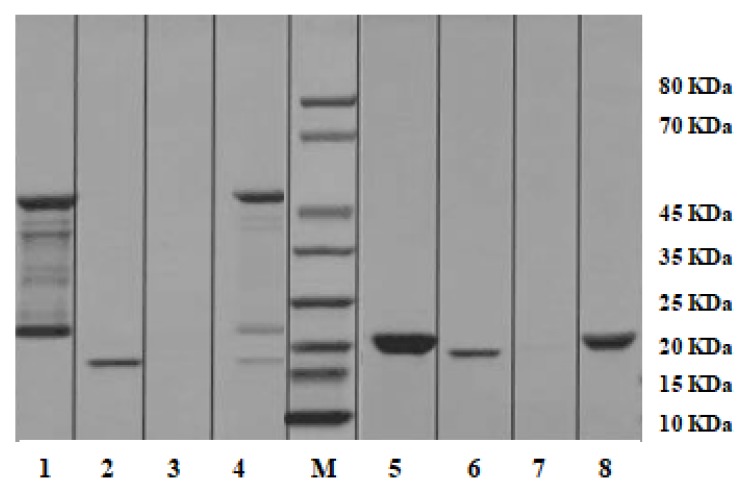
GST pull-down assays were conducted to examine the interactions between the *ATG4B* and ATG8 proteins. Lane M: protein molecular standard; lane 1: eluted protein from r*MnATG4B* incubated with r*MnATG8* under reducing conditions; lane 2: purified r*MnATG8*-His; lane 3: washing solution; lane 4: purified r*MnATG4B*-GST; lane 5: purified GST-tag protein; lane 6: purified r*MnATG8*-His; lane 7: washing solution; lane 8: eluted protein from r*MnATG8* incubated with the GST-tag under reducing conditions as a control.

To investigate the in vivo autophagy pathway involved, recombinant *ATG4B* (r*MnATG4B*) and *ATG8* (r*MnATG8*) from *M. nipponense* were successfully expressed and purified in our laboratory. GST pull-down assay was carried out to test the binding ability of recombinant *MnATG8His* and recombinant *MnATG4B-GST*, which were subjected to 12% SDS-PAGE. The experimental results demonstrated that r*MnATG8* could interact with r*MnATG4B* in *M. nipponense*, but not with the GST-tag protein (Figure 8). The molecular weight of the recombinant *MnATG4B* protein was approximately 47.05 kDa, which were mixed with glutathione-sepharose 4B resin. These results indicated that r*MnATG4B* could specifically interact with r*MnATG8*.

## 3. Discussion

According to the literature, most studies in mammalian cell lines indicates that that hypoxia positively regulates autophagy [18,19,20,21,22]. However, in vivo studies on the effects of hypoxia on expression of autophagy-related genes in crustaceans is scarce. Herein, we successfully cloned full-length cDNAs of four *ATGs* from *M. nipponense* juveniles, and classified them using phylogenetic analysis. To our knowledge, this is the first characterization of *ATG4* in prawns. The deduced *ATG4B* amino acid sequence in *M. nipponense* contains functional features typical of cysteine proteinases, including a conserved cysteine residue and Peptidase_C54 domain that are reportedly essential for catalytic activity [23,24]. Cys-74, Asp-278 and His-280 form the catalytic triad of His*ATG4B* [25], and the catalytic cleft of this enzyme recognizes *ATG8* via its regulatory loop and Trp-142 [26]. *M. nipponense ATG3* includes characteristic carboxyl-terminal, catalytic domain, and N-terminal domains, among which the N-terminal domain forms an amphipathic helix and binds membranes with sufficient curvature [27]. *M. nipponense ATG5* possesses features characteristic of canonical ubiquitin ligase enzymes, including a pair of ubiquitin-like domains, conserved calpain cleavage sites, and a helix-rich domain [28]. Autophagosome formation requires *ATG9* [29], and *M. nipponense ATG9* contains N- and C-terminal domains, and six transmembrane helices. *ATG9* forms a homodimer via dimerization between C-terminal domains, and this oligomerization is responsible for anterograde trafficking of *ATG9* to the phagophore assembly site (PAS) [30].

In the present study, all four *ATG* mRNAs were found to be expressed in multiple *M. nipponense* tissues, with higher expression levels in brain and hepatopancreas, indicating the potential for tissue-specific regulatory mechanisms, as reported previously in mammals [31,32]. The present results also revealed expression of all four *ATGs* in all prawn developmental stages, with higher levels in earlier larval stages, consistent with previous studies in insects and prawns [3,33].

Autophagy is critical for homeostasis in brain cells, and is tightly regulated under normal conditions [34]. Recent studies have demonstrated that hypoxia causes metabolic abnormalities associated with autophagy in the brain tissue of *M. nipponense* [2,3]. Therefore, we predict that autophagy may represent an adaptive response that helps to maintain cellular homeostasis following hypoxia exposure. Previous studies revealed that autophagy typically occurs in cells in response to hypoxia/reoxygenation, involving compartmentalization of stress-damaged mitochondria into autophagosomes, followed by their digestion by autolysosomes [35,36,37]. Consistent with most previous reports, our present results indicated that hypoxia activated autophagy and up-regulated mRNA levels of *ATG3*, *ATG4B*, and *ATG5*, all of which may play significant roles in autophagy-mediated regulation of cellular adaptive responses to hypoxia in *M. nipponense* brain tissue. Furthermore, we observed that *ATG4B* is localized in diverse types of neuronal cells (including secretory and astrocyte cells) in the brain; this means that it might play an important role in the cerebral ganglion of prawns in response to hypoxia. Thus, autophagy may be important for the prevention of neural degenerative diseases [2,38].

In this present work, RNA interference (RNAi) was used to probe the function of *ATG4B*. In a previous study on *M. nipponense*, we demonstrated that the effects of RNAi can last 5 days [39]. Given that the brain has a high demand for energy and higher oxygen consumption requirements than other tissues [40], and emerging evidence that autophagy serves to remove toxic materials in brain cells [41], we were curious about how hypoxia may regulate autophagy in the brain. Injection of dsRNA targeting *ATG4B* caused a significant decrease in *ATG8* transcription in the prawn brain, and decreased brain cell viability, suggesting that autophagosome biogenesis is dependent on processing of ubiquitin-like *ATG8* proteins by cysteine protease *ATG4*. There is some evidence that yeast *ATG4* is recruited to autophagosomal membranes by direct binding to *ATG8* via two evolutionarily conserved *ATG8* recognition sites [42]. Thus, the potential interactions between *ATG4B* and *ATG8* were examined. The results of the *GST* pull-down assay showed that r*ATG8* could specifically interact with r*ATG4B* (*GST* tag) in prawns. Thus, we speculated that *ATG4* functions as a constitutive *ATG8*-binding module.

## 4. Materials and Methods

### 4.1. Experimental Organisms and Hypoxia Treatment

Experimental protocols for hypoxia challenge were performed as previously reported [43]. Prawns were obtained from Dapu farm, Freshwater Fisheries Research Center (FFRC) of the Chinese Academy of Fishery Sciences. Briefly, prawns were transferred to the laboratory, acclimatized for 2 weeks, and 240 individuals (1.85 ± 0.45 g) were divided randomly into groups treated with two different dissolved oxygen (DO) concentrations (controls = 6.2 ± 0.2 mg/L; treatment = 2.0 ± 0.2 mg/L). All experiments were performed in triplicate for each group. Brain, gill, muscle, intestine, and hepatopancreas tissue (~100 mg of each from each prawn) was excised, frozen in liquid nitrogen and stored at −80 °C. Samples were also obtained from different developmental stages of larvae and post-larvae according to previously published criteria [44].

### 4.2. Full-length cDNA Cloning

Procedures for cloning *ATG3*, *ATG4B*, *ATG5*, and *ATG9A* cDNAs were performed as described previously [45]. Primers were designed based on the obtained partial cDNA sequences using the RNA-Seq database (Table 1). PCR products were purified, sequenced, sequences were subjected to comparative analysis, and MEGA4 was used for phylogenetic tree construction.

### 4.3. Expression of ATGs in Different Tissues and Developmental Stages, and Under Hypoxia Conditions

Total RNA was extracted from different tissues, from different growth stages, and from prawns cultured under control and hypoxia conditions using RNAiso Plus Reagent (TaKaRa, Dalian, China). Analysis of mRNA expression levels was performed by quantitative real-time PCR (qRT-PCR) as described with the *β-actin* housekeeping gene as an internal transcriptional reference [45]. Primers used for qPCR are listed in Table 1. Relative expression of *ATG3*, *ATG4B*, *ATG5*, and *ATG9A* was calculated using the 2^−ΔΔCt^ method [46].

### 4.4. In Situ Hybridization

The eyes and brains were dissected from the prawn, as described above, and fixed in 4% paraformaldehyde in phosphate buffer saline (PBS, pH 7.4) at 4 °C overnight. Chromogenic in situ hybridization (CISH) was performed on 4-μm thick formalin-fixed paraffin-embedded sections using the Zytofast PLUS CISH implementation kit (Zyto Vision GmBH, Bremen, Germany), as reported in a previous study [47]. The slides were dehydrated in graded alcohol solutions, air dried and mounted with DPX mountant medium (Fluka, Buchs, Switzerland). The slides were then examined under a light microscope for evaluation. The sequence 5′-GTCCCTTAGCGCACACTTCA TTCCTACACCAATCCAG-3′ for *ATG4B* was obtained and used for probing according to a previously described method [48].

### 4.5. Synthesis of Double-Stranded RNA (dsRNA) and Silencing of M. nipponense ATG4B

In vitro synthesis of dsRNA was performed as previously described [49], and 120 healthy prawns (weight = 2.4 ± 0.6 g) were assigned to experimental and control groups in triplicate. The 60 prawns in the experimental group were injected with 4 μg/g body weight *ATG4B* dsRNA via the carapace pericardial cavity membrane [49]. Controls (*n* = 60) were injected with an equal amount of dsEGFP dissolved in the injection buffer.

### 4.6. Cell Viability Assay and Biochemical Analysis

Cells were obtained from brain tissue homogenates as described previously [50], and cell viability was determined using a cell viability assay kit (G021; Nanjing Jiancheng Bioengineering Institute, Nanjing, China) according to the manufacturer’s protocol. Hexokinase (HK), pyruvate kinase (PK), and lactate dehydrogenase (LDH) activities were determined for each sample using appropriate commercial kits (Nanjing Jiancheng Bioengineering Institute). The protein concentration of enzyme extracts was determined using the Bradford method [51], and all enzyme assays were performed in quadruplicate.

### 4.7. Pull-down Analysis

Recombinant *ATG4B* (*MnATG4B*) and *ATG8* (*MnATG8*) from *M. nipponense* were expressed in *E. coli* as His-tagged fusion proteins using the pET-28a (Novagen, Darmstadt, Germany) expression system, as described in our previous study. *MnATG4B* and *MnATG8* were purified using the *GST*-Bind resin. The pull-down assay was performed as previously described [52]. After the proteins were washed thoroughly with 12 mL washing buffer (0.5 M NaCl, 60 mM imidazole, 20 mM Tris HCl [pH 7.9]), they were eluted with 6 mL elution buffer (0.5 M NaCl, 1 M imidazole, 20 mM Tris HCl [pH 7.9]) and analyzed with 12% SDS-PAGE. The *GST* protein was used as a negative control in this assay.

### 4.8. Statistical Analysis

Results are presented as the mean ± standard error. Statistical analysis was performed using Statistical Package for the Social Sciences (version 19.0). Different tissues and developmental stages were compared using one-way analysis of variance and Duncan’s multiple range tests. Treatment and control groups were independently compared with Student’s t-tests, and *p* < 0.05 was considered statistically significant.

## 5. Conclusions

In summary, we cloned and characterized full-length cDNAs of *ATG3*, *ATG4B*, *ATG5*, and *ATG9A* from *M. nipponense*, and determined their expression profiles in different tissues and developmental stages. Hypoxia up-regulated the expression of the autophagy-related genes *ATG3*, *ATG4B*, and *ATG5* at the transcriptional level in prawn brain tissue. In vivo silencing of the AG4B gene to prevent autophagy resulted in a decrease in brain cell viability, and recombinant *MnATG4B* exhibited binding activity with the r*MnATG8* protein. These findings reveal that the fundamental mechanisms of autophagy involve putative adaptive catabolic processes that are activated in response to hypoxia.

## Figures and Tables

**Table 1:** Primers used in this study.

Primer	Primer Sequence (5′ to 3′)
*ATG3*-R1 (5′ RACE out primer)	TAAAACACCAGTCTCC
*ATG3*-R2 (5′ RACE in primer)	GGACTCCTTGAGGACCGG
*ATG3*-R3 (5′ RACE in primer)	CTACACCTCGTGCTTTGC
*ATG3*-F1 (3′ RACE out primer)	GGAAACCCACCCAAATGTGACAGG
*ATG3*-F2 (3′ RACE in primer)	CTGTGTTAGAGGGTGGTGGTGAGC
*ATG4B*-R1 (5′ RACE out primer)	TCCGACGATAAGTGAG
*ATG4B*-R2 (5′ RACE out primer)	CTGTGCAAGATCTGTCTG
*ATG4B*-R3 (5′ RACE out primer)	TTGAATAACGCCGTCCTA
*ATG4B*-F1 (3′ RACE out primer)	TGCTCTTTTGCTCAACGGATGCCT
*ATG4B*-F2 (3′ RACE in primer)	AACAATTGGACCCCTCTTTGGCAC
*ATG5*-R1 (5′ RACE out primer)	TATGAATCTGGAGCAC
*ATG5*-R2 (5′ RACE out primer)	AGCTGGACACAGACTGGG
*ATG5*-R3 (5′ RACE out primer)	GACCATCCCATATCTCGC
*ATG5*-F1 (3′ RACE out primer)	TAATGGAAGGTGACCGAATTGTGA
*ATG5*-F2 (3′ RACE in primer)	CCCTGGATACACCAGTACAATGGC
*ATG9A*-R1 (5′ RACE out primer)	TAGAGTGCGTGTCGGG
*ATG9A*-R2 (5′ RACE out primer)	TCCTCCCTCTCTCTGTCC
*ATG9A*-R3 (5′ RACE out primer)	CCCTGGTGTCATTCCCAC
*ATG9A*-F1 (3′ RACE out primer)	GCTCCAAGTTCCACGGTGCCATCA
*ATG9A*-F2 (3′ RACE in primer)	GCAAATCTCACAACGATGCCCCCA
*ATG3*-F (Real-Time primer)	ACCCAAATGTGACAGGTCCT
*ATG3*-R (Real-Time primer)	TCACCACCACCCTCTAACAC
*ATG4B*-F (Real-Time primer)	ACTTCAGACAAGGGATGGGG
*ATG4B*-R (Real-Time primer)	AAGCTGTCCATACCCAGTCC
*ATG5*-F (Real-Time primer)	TGGTTCCAAGGCTGTCGTAT
*ATG5*-R (Real-Time primer)	AACCACATTTCTGCGTCCTG
*ATG9A*-F (Real-Time primer)	CCTTGGTGTGGGAATATGCG
*ATG9A*-R (Real-Time primer)	AGCTTTCTCTCGCCAGTGAT
*β-Actin*-F (Real-Time primer)	TATGCACTTCCTCATGCCATC
*β-Actin*-R (Real-Time primer)	AGGAGGCGGCAGTGGTCAT
ds*ATG4B*-F (RNAi)	TAATACGACTCACTATAGGGGACAGATGGTGCTTGCAGAA
ds*ATG4B*-R (RNAi)	TAATACGACTCACTATAGGGTTCATCCCCCATAAAACCAA

## Abbreviations

qPCR	Quantitative real-time reverse transcription PCR
ATG	Autophagy-related genes
ORF	Open reading frame
ROS	Reactive oxygen species
mRNA	Messenger RNA
ISH	In situ hybridization
dsRNA	Double-stranded RNA
cDNA	Complementary DNA
FFRC	Freshwater Fisheries Research Center
SDS	Sodium dodecyl sulfate
PAGE	polyacrylamide gel electrophoresis
HK	Hexokinase
PK	Pyruvate kinase
LDH	Lactate dehydrogenase
